# 1,25-dihydroxyvitamin D3 improves non-alcoholic steatohepatitis phenotype in a diet-induced rat model

**DOI:** 10.3389/fendo.2025.1528768

**Published:** 2025-03-21

**Authors:** Mei Liu, Xiang-Zhun Song, Liu Yang, Yu-Hui Fang, Liu Lan, Jing-Shu Cui, Xiao-Chen Lu, Hai-Yang Zhu, Lin-Hu Quan, Hong-Mei Han

**Affiliations:** ^1^ Department of Gastroenterology, Affiliated Hospital of Yanbian University, Yanji, Jilin, China; ^2^ Department of Gastroenterology, Jilin Provincial People’s Hospital, Changchun, Jilin, China; ^3^ Department of Gastroenterology and Hepatology, Characteristic Medical Center of the Chinese People’s Armed Police Force, Tianjin Key Laboratory of Hepatopancreatic Fibrosis and Molecular Diagnosis & Treatment, Tianjin, China; ^4^ Department of Dermatology, Fuyang People’s Hospital of Anhui Medical University, Fuyang, Anhui, China; ^5^ Department of Pathology, Affiliated Hospital of Yanbian University, Yanji, Jilin, China; ^6^ Department of Gastroenterology, Jimo District People’s Hospital, Qingdao, Shandong, China; ^7^ Department of College of Pharmacy, Yanbian University, Yanji, Jilin, China

**Keywords:** vitamin D3, non-alcoholic steatohepatitis, CYP450, inflammation, macrophage

## Abstract

We studied the potential protective effects of 1,25-dihydroxyvitamin D3 (1,25 VD3) supplementation on liver damage induced by a choline-deficient (CD) diet in rats, where impaired liver function leads to decreased 25-hydroxyvitamin D3 levels, the precursor for the active 1,25 VD3. The CD diet reduced serum 25 VD3 levels and increased liver enzymes, indicative of liver damage. Conversely, 1,25 VD3 supplementation alleviated liver damage, reducing liver enzymes and improving histopathological features characteristic of non-alcoholic steatohepatitis (NASH). Oxidative stress and inflammation were mitigated by 1,25 VD3, as evidenced by decreased malondialdehyde and nuclear factor kappa B (NF-κB) expression, and increased total antioxidant capacity (TAOC). 1,25 VD3 also enhanced fatty acid metabolism by increasing peroxisome proliferator-activated receptor alpha (PPARα) and carnitine palmitoyltransferase-1 (CPT-1) expression, promoting lipid transport and oxidation. Additionally, 1,25 VD3 supplementation modulated inflammation by increasing PPARγ expression, reducing NF-κB expression, and decreasing pro-inflammatory cytokines (TNF-α, IL-1β). Anti-inflammatory cytokines (IL-10, IL-4) were increased, and macrophage polarization was shifted towards an anti-inflammatory M2 phenotype. Moreover, 1,25 VD3 upregulated CYP2J3, a cytochrome P450 epoxygenase that converts arachidonic acid to anti-inflammatory epoxyeicosatrienoic acids (EETs) and decreased soluble epoxide hydrolase activity, likely contributing to increased EET levels. Correlation studies revealed positive associations between 1,25 VD3 supplementation, CYP2J3 expression, EETs, as well as negative correlations with NF-κB and TNF-α. PPARα expression positively correlated with TAOC and CPT-1, while PPARγ expression negatively correlated with inflammatory markers. These findings demonstrate the therapeutic potential of 1,25 VD3 in alleviating NASH through regulation of fatty acid metabolism, inflammation, and oxidative stress.

## Introduction

Non-alcoholic fatty liver disease (NAFLD), proposed to be renamed metabolic (dysfunction)-associated fatty liver disease (MAFLD) (1) is a common chronic liver condition marked by excessive lipid accumulation in hepatocytes, independent of alcohol consumption or other toxic factors (2). It encompasses a spectrum of progressive stages, including simple non-alcoholic fatty liver, non-alcoholic steatohepatitis (NASH), liver cirrhosis, and hepatocellular carcinoma, highlighting its clinical significance and potential for progression to hepatocarcinogenesis. The pathogenesis of NASH is marked by hepatic lipid peroxidation and inflammation (3–5). Recently, active vitamin D3 (1,25-dihydroxyvitamin D3, (1,25 VD3) has emerged as a potential therapeutic agent, exhibiting antioxidant and anti-inflammatory properties. Notably, studies have shown that NASH patients have lower serum 1,25 VD3 levels compared to healthy controls (6, 7). Moreover, 1,25 VD3 supplementation has been found to improve insulin resistance and liver enzyme levels in NASH patients (6–10). Animal models have replicated NASH-like liver histopathology using a choline-deficient, amino acid-defined (CDAA) diet (11). Our previous research demonstrated that 1,25 VD3 improves lipid peroxidation and inflammation in NASH-induced rats in a dose-dependent manner (12). However, the specificity and mechanisms underlying this effect remain unclear, warranting further investigation. Recent research has highlighted the significance of the cytochrome P450 (CYP450) pathway in arachidonic acid (AA) metabolism in the development of non-alcoholic steatohepatitis (NASH) (13). Specifically, studies have identified substantial changes in CYP450 metabolites and AA metabolism in patients with NASH (14) and in animal models with NASH induced by high-fat diets (15, 16). The CYP450 pathway produces epoxide eicosatrienoic acids (EETs) as primary metabolites, comprising 5,6-EET, 8,9-EET, 11,12-EET, and 14,15-EET (17). Notably, EETs have been extensively shown to possess anti-lipid peroxidation and anti-inflammatory properties across various diseases (18–20). However, the beneficial effects of EETs are compromised upon hydrolysis by soluble epoxide hydrolase (sEH), converting them to dihydroxy eicosatrienoic acids (DHETs) (21). Interestingly, while EETs exhibit anti-inflammatory activities, the role of DHETs remains controversial.

The anti-inflammatory and antioxidant effects of EETs in the liver are primarily mediated through the peroxisome proliferator-activated receptor-alpha (PPARα) and nuclear factor-kappaB (NF-κB) pathways. As potent activators of PPARα (22), EETs regulate lipid homeostasis and mitigate lipid peroxidation in the liver (23–25). Studies employing mouse models of NAFLD induced by high-fat diets (HFD) have demonstrated the therapeutic potential of EETs. For instance, administering 14,15-EET to CYP450 2J2 (CYP2J2)-overexpressing mice reduced NF-κB expression, lipid peroxidation, and inflammation in the liver. *In vitro* experiments using palmitic acid-treated HepG2 cells further revealed that 14,15-EET inhibited the NF-κB/JNK signaling pathway, decreased malondialdehyde (MDA) levels, and enhanced antioxidant enzyme activities, including superoxide dismutase, catalase, and glutathione peroxidase (26). Consistent with these findings, other studies have shown that elevated EET levels alleviate liver inflammation in mice with NASH induced by HFD and methionine-choline-deficient (MCD) diets. Specifically, EETs suppressed the NF-κB pathway, leading to reduced liver inflammation (14, 15, 27). These studies collectively highlight the protective role of EETs in liver disease. To further explore the mechanisms underlying 1,25 VD3’s therapeutic effects, we established a rat model of NASH using a choline-deficient, amino acid-defined (CDAA) diet and supplemented 1,25 VD3. We measured metabolic proteins, metabolites, and key enzymes of the liver CYP450 pathway to investigate whether 1,25 VD3 improves lipid peroxidation and inflammation through the CYP450 pathway.

## Materials and methods

### Materials

Standard chow diet (choline-sufficient, amino acid-defined, Cat # TP 1R810) and CDAA diet (Cat # TP 1R800) were procured from Trophic Animal Feed High-Tech Company, China. 1,25 VD3 from Sigma-Aldrich (CAS # 128723-16-0), BCA protein quantitation kit (Boster Bio, Cat # AR0146), RIPA lysis buffer (Boster Bio, Cat # 0105), protease inhibitor cocktails (Boster, Bio Cat # AR1182), phosphatase inhibitor (Boster Bio, Cat # AR1183), color pre-dyed protein marker (Boster Bio, Cat # AR1113), Western-specific primary and secondary antibody diluent (Boster Bio, Cat # AR1017), wash buffer TBS-T (Boster Bio, Cat # AR0195-10), ECL chemiluminiscent reagent (Boster Bio, Cat # AR1196), BSA TBS buffer system blocking solution (Boster Bio, Cat # AR0189), NF-κB antibody (Boster Bio, Cat # A01228-1), β-actin antibody (Boster Bio, Cat # M01263), HRP-conjugated goat anti-rabbit IgG (Boster Bio, Cat # BA1054), CD163 antibody (Boster Bio, Cat # A00812-2), CD11c antibody (Boster Bio, Cat # A00357-3), CD68 antibody (Boster Bio, Cat # BA3638), fluorescent (DyLight 488) labelled goat anti-rabbit IgG (Boster Bio, Cat # BA1127), fluorescent (DyLight 594) labelled goat anti-rabbit IgG (Boster Bio, Cat # BA1142), rat TNF-α ELISA kit (Jiangsu Enzyme Immunoassay Co., Ltd., Cat # MM-0180R1), rat IL-1β ELISA kit (Boster Bio, Cat # EK0393), rat IL-4 ELISA kit (Jiangsu Enzyme Immunoassay Co., Ltd, Cat # MM-0191R1), rat IL-10 ELISA kit (Boster Bio, Cat # EK0418), 25 VD3 assay kit (Roche Diagnostics, Cat # 07028148190), TAOC assay kit (Nanjing Jiancheng, Cat # A015-3-1), MDA assay kit (Nanjing Jiancheng, Cat # A003-1-2), and free fatty acid kit (Kunchuang Biotechnology, Xian, China, Cat # SK125-2).

### Animal study design and experimental procedures

The experimental procedures for this study complied with the ethical standards of the China Experimental Animal Management Association and were approved by the Ethics Committee of Yanbian University (approval number YD202309110024). Based on our previous research (12), we conducted a 12-week study using 6-week-old, specific-pathogen-free-grade Wistar rats purchased from Changchun Yisi Animal Co., Ltd.

**Figure 1:**
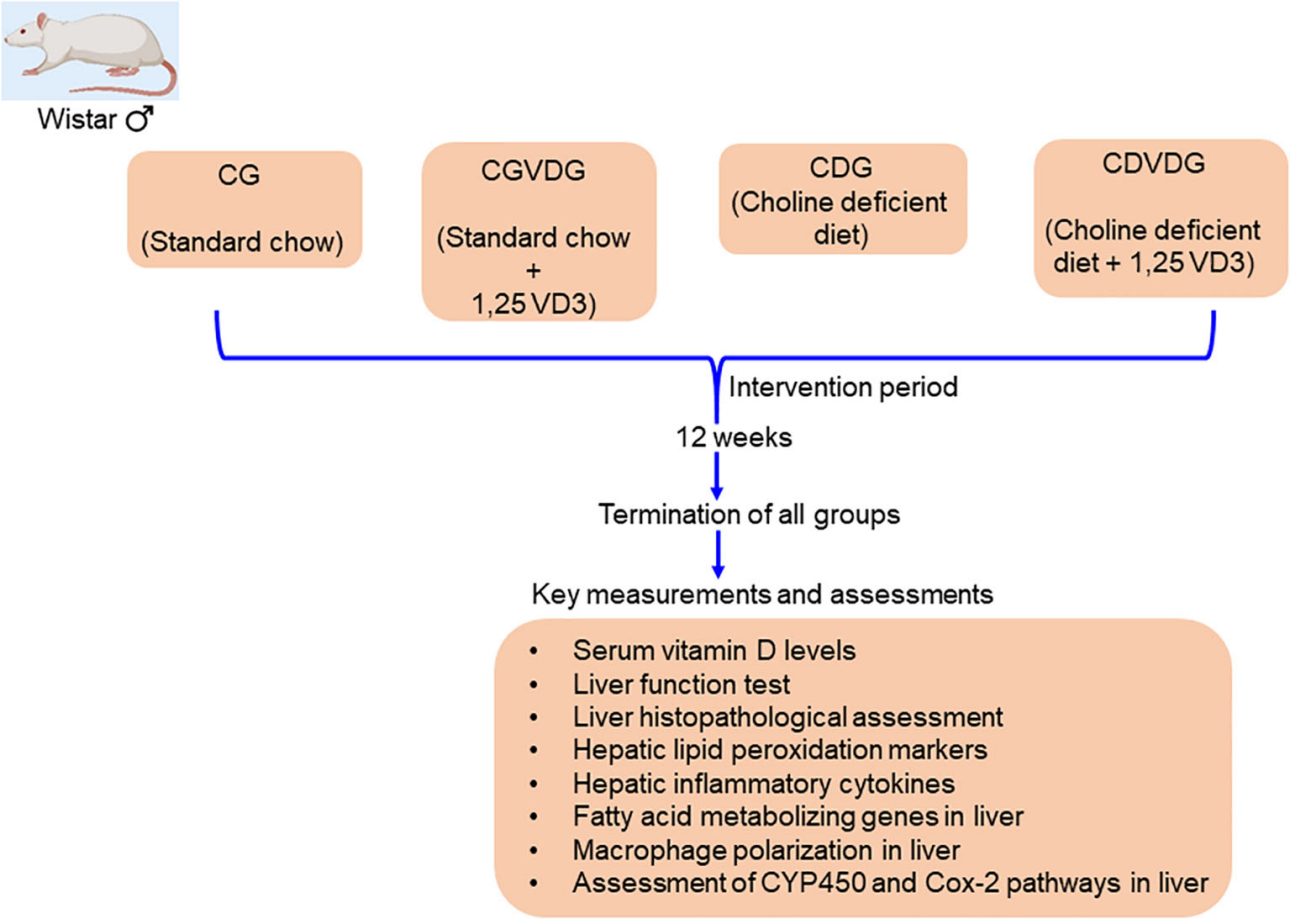
A schematic diagram representing study design.

Following an adaptation period, the rats were divided into four groups: 1. Control Group (CG): fed a normal rat chow diet; 2. Control plus 1,25 VD3 supplement Group (CVDG): fed a normal rat chow diet with 1,25 VD3 injection; 3. Choline-deficient Group (CDG): fed a CD diet that was amino acid sufficient; and 4. Choline-deficient plus 1,25 VD3 supplement Group (CDVDG): fed a CDAA diet with 1,25 VD3 injection. (**Figure 1** presents the schematic diagram of the experimental design). We administered 5μg 1,25 VD3/kg body weight via intraperitoneal injection twice a week (12).

To prepare the 1,25 VD3 solution, 1mg 1,25 VD3 powder (Sigma Reagent Company) was dissolved in 20μL anhydrous ethanol and diluted in 0.9% sodium chloride to make a 5μg/mL solution. This solution was stored in a dark environment at 4°C and prepared every 2 weeks. Rats in the CDG received an equivalent volume of anhydrous ethanol in 0.9% sodium chloride (1mL/kg body weight) intraperitoneally. After 12 weeks of treatments, the rats were fasted for 2 h and euthanized by cervical dislocation. Body weight was measured, and blood was collected from the abdominal aorta. Serum was separated by centrifugation at 4°C (3000 rpm). The liver was removed, and its wet mass was recorded. A portion of the right lobe was fixed with 4% formaldehyde for histological analysis, while the remaining tissue was homogenized for further analysis.

### Liver enzymes and blood lipids

Serum aspartate transaminase (AST) and alanine transaminase (ALT) activities were measured using a cobas c702 automatic biochemical analyzer (Roche Diagnostics GmbH). Serum triglyceride (TG), total cholesterol (TC), high-density lipoprotein-cholesterol (HDL-C), and low-density lipoprotein-cholesterol (LDL-C) levels were determined using kits from Nanjing Jiancheng Bioengineering Institute, following the manufacturer’s instructions.

### Histopathological evaluation

Liver tissue from the right lobe was fixed with 4% formaldehyde, embedded in paraffin, and stained with haematoxylin and eosin (H&E). Steatosis, activity, and fibrosis (SAF) were scored by two pathologists blinded to the study design. Five visual fields from each section were magnified 200 times and averaged for statistical analysis. The SAF score was based on NASH Clinical Research Network criteria (22, 23) for grading steatosis and activity (**Table 1**). In this unweighted scoring system (28), the activity score is the sum of the lobular inflammation and hepatocellular ballooning scores, ranging from 0 to 5. A higher score indicates greater disease activity. A SAF score ≥3 was diagnosed as NASH.

**Table 1:** NASH scoring.

Steatosis		
Grade	Steatosis percentage (hepatocytes with fat droplets)	Description
0	<5%	No significant steatosis
1	5–33%	Mild steatosis
2	34–66%	Moderate steatosis
3	>66%	Severe steatosis

Lobular inflammation		
Score	Description	
0	No inflammatory foci	
1	<2 inflammatory foci per 200x field	
2	2–4 inflammatory foci per 200x field	
3	>4 inflammatory foci per 200x field	

Ballooning		
0	None	
1	Few ballooned cells	
2	Many ballooned cells or prominent ballooning	

### Serum 25 VD3 measurement

Serum 25 VD3 levels were determined using the Roche electrochemiluminescence method on a Cobas 8000 automatic biochemical immunity analyzer (Roche Diagnostics GmbH).

### Oxidation status

Liver lipid peroxidation was evaluated by measuring malondialdehyde (MDA) levels using a thiobarbituric acid kit, while total antioxidant capacity (TAOC) was assessed via the ferric reducing antioxidant power (FRAP) method, both performed according to the manufacturer’s instructions (Nanjing Jiancheng Bioengineering Institute).

### Detection of PPARα and CPT-1 by qPCR

PPARα regulates lipid homeostasis and liver lipid peroxidation (26, 27, 29). PPARα stimulates liver expression of carnitine palmitoyltransferase-1 (CPT-1), initiating mitochondrial fatty acid transport for β-oxidation (30). To investigate the expression of PPARα and CPT-1, we measured their mRNA levels in liver tissue using qPCR. Total RNA (20μg) was extracted from liver tissue using Trizol solution (Invitrogen). Reverse transcription was performed using RevertAid Reverse Transcriptase (Thermo Scientific). Quantitative PCR amplification was then carried out. qPCR data were processed using the ΔΔCT method. Primer sequences are listed in **Table 2**.

**Table 2:** qPCR primers’ sequence.

Gene	Sequence	Seq/RefSeq
PPARα(155bp)	F:CGGGTCATACTCGCAGGAAAG R:TGGCAGCAGTGGAAGAATCG	NM_013196.2
CTP-1(141bp)	F:CTGACGCCCGAGTTCCTG R:GCCTTCTGTCCTCTGTGTGG	NM_078622
PPARγ(141bp)	F:CCTTTACCACGGTTGATTTCTC R:CAGGCTCTACTTTGATCGCACT	NM_013124.3
CYP2J3(75bp)	F:TTCAGAATGTCCGTCACCAT R:TTCCTCTTCGACATCACAGC	NM_175766
GAPDH(114bp)	F:TTCAACGGCACAGTCAAGG R:CTCAGCACCAGCATCACC	NM_017008.4

### Inflammation and macrophage polarization analysis in liver tissue

To investigate inflammation and macrophage polarization, we measured: 1. NF-κB levels in liver tissue using Western blotting; 2. M1 (CD68+CD11c+) and M2 (CD68+CD163+) macrophage populations using double immunofluorescence labelling; 3. Pro-inflammatory (TNF-α and IL-1β) and anti-inflammatory (IL-10 and IL-4) factor levels using enzyme-linked immunosorbent assay (ELISA); and 4. PPARγ mRNA expression in liver tissue using qPCR.

For macrophage polarization study, we used the double immunofluorescence method. Liver tissue sections were dewaxed, rehydrated, and incubated with primary antibodies (anti-CD68, 1:100; anti-CD11c, 1:100; or anti-CD163, 1:200) followed by secondary antibodies labeled with fluorescein. DAPI staining and fluorescence microscopy were used to visualize and count macrophages. M1 macrophages were identified as CD68+CD11c+ cells, while M2 macrophages were identified as CD68+CD163+ cells. Nuclei were counterstained with DAPI (blue) for cell identification. The M1/M2 ratio was determined by counting CD68+CD11c+ (M1) cells and dividing it by the number of CD68+CD163+ (M2) cells in the same fields. Two pathologists randomly selected six regions with high positive staining rates from each group of double-stained liver tissue sections. The cell counts were averaged for each animal before calculating group means and conducting statistical analysis.

### Western blotting

Liver tissue samples (100mg) were homogenized in RIPA buffer supplemented with protease and phosphatase inhibitors. The lysates were centrifuged at 14,000 × g for 15 minutes at 4°C to remove debris, and the supernatant was collected for protein quantification using the BCA assay. Equal amounts of protein (30 µg per lane) were loaded onto a 10% SDS-PAGE gel and electrophoresed at 100 V. Proteins were then transferred to a PVDF membrane at 300 mA for 1.5 hours in transfer buffer. The membrane was blocked in 5% non-fat milk in TBST for 1 hour at room temperature and then incubated overnight at 4°C with an antibody against NF-κB (1:1000 dilution). After washing, the membrane was incubated with an HRP-conjugated secondary antibody (1:2000 dilution) for 1 hour at room temperature. Signal detection was performed using ECL reagents (Boster Bio-Engineering Limited Company). Bands were visualized using a chemiluminescent imaging system. The membranes were stripped and reprobed with an antibody against β-actin (1:1000 dilution) as a loading control, followed by incubation with an HRP-conjugated secondary antibody (1:2000 dilution). Densitometry was performed using ImageJ software, and NF-κB expression was normalized to β-actin.

### Analysis of CYP450 pathway

CYP2J3 expression was assessed for expression by qPCR following the protocol described above. To quantify the metabolites of the liver CYP450 pathway, specifically (EETs) and DHETs, we employed targeted liquid chromatography-tandem mass spectrometry (LC-MS/MS). Sample preparation was performed by adding PBS containing 0.1% butylated hydroxytoluene and an isotope internal standard to the sample. The mixture was then ground, incubated at 4°C for 1 hour, and centrifuged. The resulting supernatant was extracted and subsequently enriched with AA using an Oasis MAX SPE column (Waters, USA). Quantitative analysis of eicosanoids was performed after solid-phase extraction-enrichment in electrospray ionization mode using an Exion UPLC-QTRAP 6500 PLUS system (Sciex) (19, 20).

### Statistical analysis

All experiments were repeated three times, with the average result taken for analysis. All data were analyzed by IBM SPSS Statistics 25.0 statistical software, and a two-way analysis of variance was used to explore the effects of 1,25 VD3 and NASH status. If statistical differences were detected, between-group comparisons were carried out using the Fisher’s Least Significant Difference test. Pearson’s correlation coefficient was used to analyze the correlation between parameters. Data are expressed as means and standard error of the mean (SEM); results were considered statistically significant when *p*< 0.05.

## Results

### 1,25 VD3 improves liver function and alleviates histopathological features of CDAA-induced NASH

**Figure 2:**
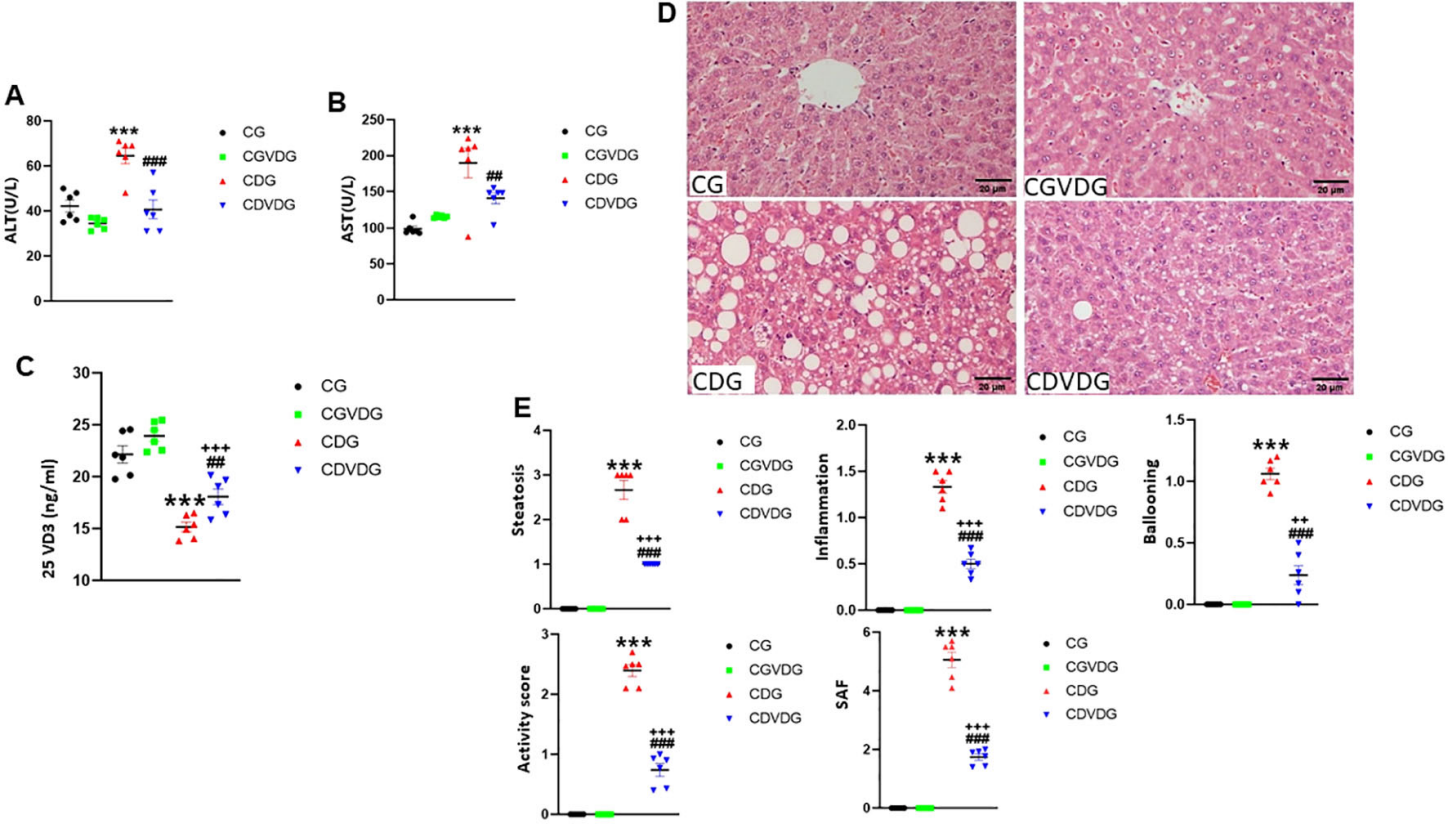
Effects of 1,25 VD3 supplementation on serum biomarkers and liver histopathology in a rat model of NASH. **(A)** Serum ALT and **(B)** AST activities were measured using Automatic Biochemical Analyzer. **(C)** 25VD_3_ levels were measured using Roche Electrochemiluminescence method in the indicated groups. **(D)** Representative H&E-stained liver sections (5 μM) from the indicated groups. **(E)** Histopathological scores for steatosis, lobular inflammation, ballooning degeneration, activity, and SAF scores were determined following the protocol as described in **Table 1**. Data are presented as mean ± SEM, n=6. Statistical significance: A-C - ***p < 0.001 vs. CG; ##p < 0.01, ###p < 0.001 vs. CDG; +++p < 0.001 vs. CDVDG. D-E - ***p <0.001 vs. CG; ###p < 0.001 vs. CDG; ++p < 0.01, +++p < 0.001 vs. CDG. CG: Normal rat chow diet; CVDG: Normal diet + 1,25 VD3; CDG: Choline-deficient diet; CDVDG: Choline-deficient diet + 1,25 VD3.

To investigate the impact of 1,25 VD3 supplementation on liver function in the CDAA-induced NASH model, we assessed key liver function markers by measuring serum ALT (**Figure 2A**) and AST (**Figure 2B**) activities. Compared to the CG, the CDG showed elevated serum AST (p < 0.001) and ALT (p < 0.001) levels, indicating impaired liver function. Conversely, CDVDG demonstrated reduced AST and ALT levels compared to CDG (p < 0.001), suggesting improved liver health. Notably, CDVDG and CGVDG groups exhibited similar AST and ALT levels. Increased 25 VD3 levels in CDVDG versus CDG indicate enhanced liver function, as the liver produces this precursor to active 1,25 VD3 (**Figure 2C**). These findings imply that 1,25 VD3 supplementation protects against CDAA-induced impairment in liver function.

The hallmark histopathological features of NASH, including steatosis, inflammation, and hepatocyte injury, were assessed using H&E staining (**Figure 2D**). Analyses of histopathological changes (**Figure 2E**) revealed that the CDG group had significantly increased steatosis, lobular inflammation, ballooning degeneration, activity score, and SAF score compared to the CG (all p < 0.001). The CDVDG demonstrated significantly reduced steatosis, lobular inflammation, ballooning degeneration, activity score, and SAF score compared to the CDG (all p < 0.001). These results suggest that 1,25 VD3 supplementation alleviates liver damage in CDAA diet-induced NASH rats, improving steatosis, inflammation, and histopathological scores.

### 1,25 VD3 attenuates oxidative stress and enhances fatty acid metabolism in CDAA-induced NASH

**Figure 3:**
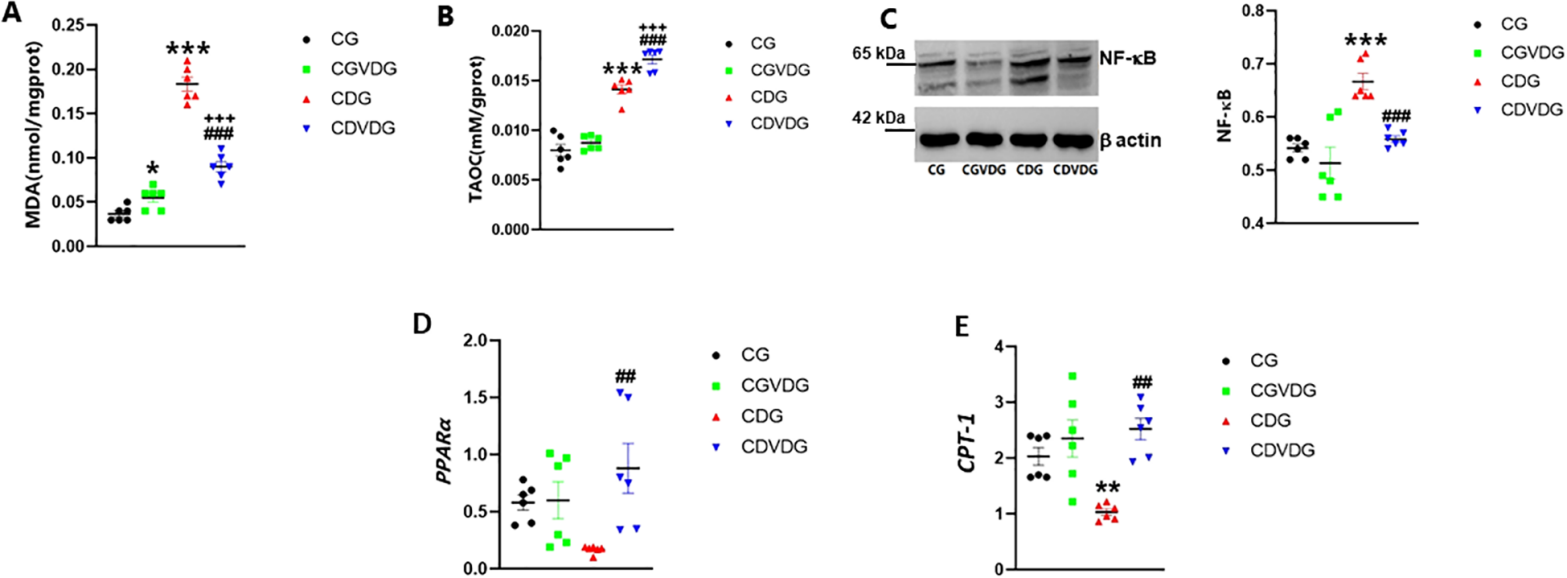
Effects of 1,25 VD3 supplementation on hepatic lipid peroxidation, inflammation and fatty acid metabolizing genes in NASH rats. To assess oxidative stress, hepatic **(A)** MDA was measured using a thiobarbituric acid kit and **(B)** TAOC levels measured using the FRAP method. **(C)** NF-κB expression in liver tissue, analyzed by Western blotting and normalized to β-actin (left panel showing representative blot and right panel showing quantification by densitometry of 65 kDa band). Antibodies used and their dilutions: primary antibodies against NF-κB (anti-rabbit, 1:1000) and β-actin (anti-rabbit, 1:1000), followed by HRP-conjugated secondary antibody (1:2000). **(D)** PPARα and **(E)** CPT-1 mRNA levels were quantified by qPCR in liver tissues of indicated groups, and expressed as relative mRNA levels following normalization to GAPDH mRNA levels. Data are presented as mean ± SEM, n=6. *p < 0.05, **p<0.01 and ***p < 0.001 vs CG; ##p < 0.01 and ###p < 0.001 vs CDG.

Liver MDA and TAOC levels were evaluated to investigate oxidative stress in NASH. While MDA levels remained higher in the CDVDG than in the CGVDG (p < 0.001), 1,25 VD3 supplementation reduced MDA levels in the CDVDG compared to the CDG (p < 0.001) (**Figure 3A**). CGVDG showed a modest yet significant increase in MDA levels compared to the CG. TAOC levels were significantly higher in the CDG and CDVDG compared to their respective controls (CG and CGVDG, p < 0.001). Notably, TAOC levels in the CDVDG surpassed those in the CDG (p < 0.001), while no difference was observed between the CGVDG and CG (**Figure 3B**).

Given that oxidative stress and inflammation are closely linked, we examined NF-κB expression, a key regulator of inflammatory pathways. The CDAA diet significantly increased NF-κB expression in the CDG compared to the CG rats (p < 0.001). The upregulation was observed as an increased intensity of the 65 kDa NF-κB band. Supplementation with 1,25 VD3 significantly mitigated the CDAA diet-induced increase in NF-κB expression observed in CDG rats (p < 0.001). In the CDVDG, NF-κB levels were restored to values comparable to the CG (p < 0.001) (**Figure 3C**).

NF-κB-driven inflammation in NASH can impact fatty acid metabolism, critically modulated by PPARα and CPT-1. PPARα regulates lipid homeostasis and liver lipid peroxidation (26, 27, 29), and also stimulates liver expression of CPT-1, initiating mitochondrial fatty acid transport for β-oxidation (30). PPARα and CTP-1 expression were significantly higher in the CDVDG compared to the CDG (p < 0.001 and p < 0.01, respectively). No significant changes in PPARα and CTP-1 expression were observed between the CGVDG and CG (**Figures 3D, E**).

These results demonstrate that the CDAA diet increases liver MDA and TAOC levels, while 1,25 VD3 supplementation enhances PPARα and CTP-1 expression, reduces MDA, and increases TAOC, indicating potential anti-lipid peroxidation and antioxidation activity.

### 1,25 VD3 favorably modulates KC polarization and upregulates PPARγ in NASH

**Figure 4:**
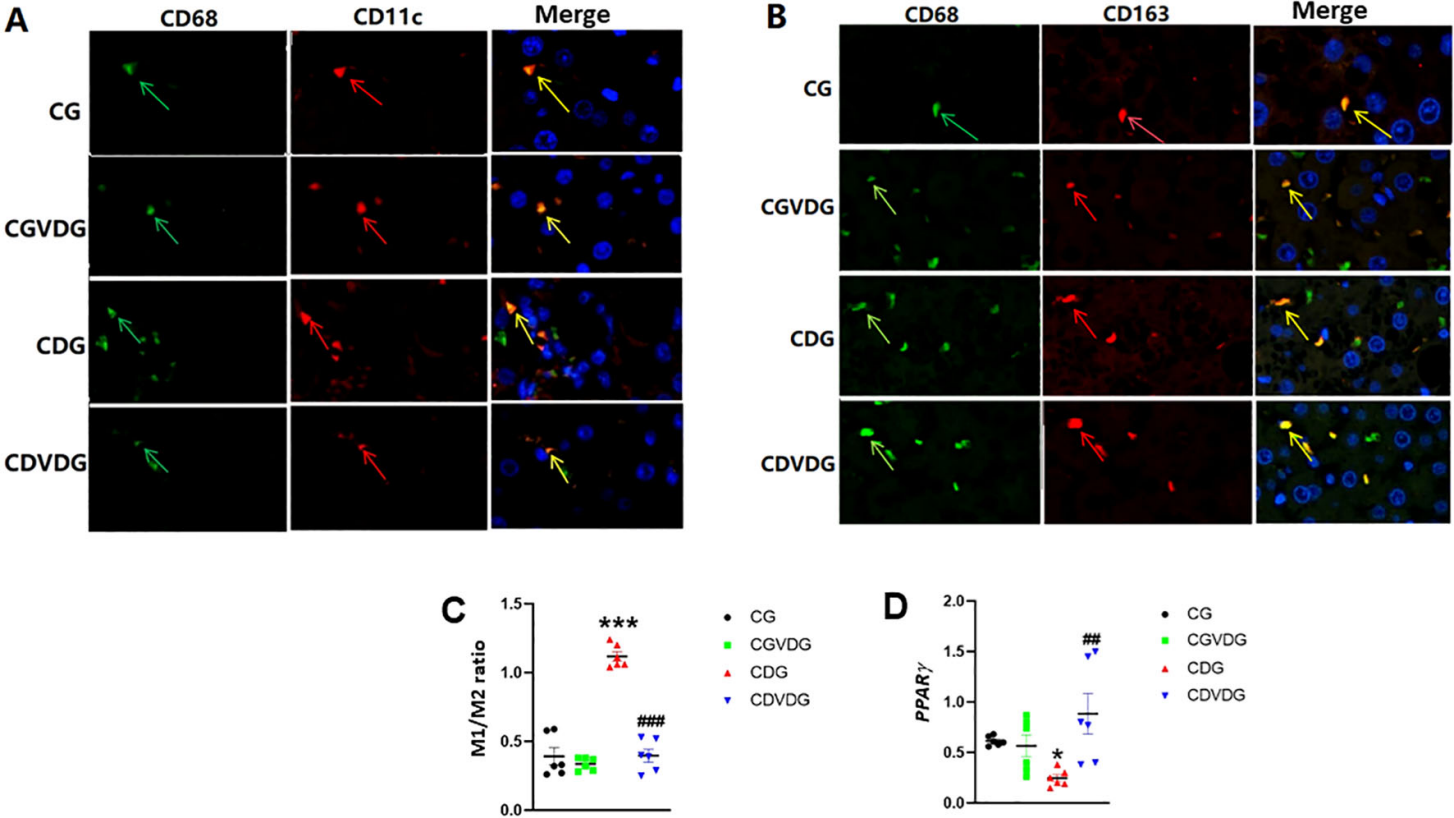
Effects of 1,25 VD3 supplementation on macrophage polarization and PPARγ expression in NASH rats. **(A)** M1-type KCs identified by double immunofluorescence staining; green arrows indicate CD68-positive cells, red arrows indicate CD11c-positive cells, and yellow arrows indicate CD11c/CD68 double-positive cells (M1KCs). **(B)** M2-type KCs identified by double immunofluorescence staining; green arrows indicate CD68-positive cells, red arrows indicate CD163-positive cells, and yellow arrows indicate CD163/CD68 double-positive cells (M2KCs). Antibodies were diluted as follows: anti-CD68 (1:100), anti-CD11c (1:100), and anti-CD163 (1:200). **(C)** The M1/M2 macrophage ratio was determined by quantifying the proportion of M1 and M2 KCs in liver tissue, as identified through double immunofluorescence staining. **(D)** PPARγ levels in liver tissue of the indicated groups were determined by qPCR and expressed as relative mRNA levels following normalization to GAPDH mRNA levels. Data are presented as mean ± SEM, n=6. *p < 0.05 and ***p < 0.001 vs CG; ##p < 0.01 and ###p < 0.001 vs CDG.

KCs, including M1 and M2 macrophages, contribute to liver inflammation in NASH. An imbalance between pro-inflammatory M1 and anti-inflammatory M2 macrophages worsens inflammation. NF-κB upregulation favors M1 macrophage differentiation over M2, amplifying inflammatory responses in KCs. CD11c-positive, CD68-positive cells (yellow or orange in merged images) indicate a pro-inflammatory M1 phenotype (**Figure 4A**). CD68-positive, CD163-positive cells indicate an anti-inflammatory M2 phenotype (**Figure 4B**). The M1 (pro-inflammatory)/M2 (anti-inflammatory) ratio in the CDG was significantly higher than in the CG (*p*< 0.001), but the M1/M2 ratio in CDVDG was not significantly different from the CGVDG (*p*> 0.05). Compared with the CG, the M1/M2 ratio in the CGVDG was not significantly different, but was significantly lower in the CDG (*p*< 0.001) (**Figure 4C**).

Since PPARγ activity negatively regulates M1/M2 ratio and liver inflammation (31), we evaluated its gene expression in liver. PPAR-γ expression was significantly downregulated in CDG (p < 0.01) but markedly upregulated in CDVDG, exceeding CG levels (p < 0.01) (**Figure 4D**).

### 1,25 VD3 restores cytokine balance by reducing pro-inflammatory and enhancing anti-inflammatory mediators in NASH

**Figure 5:**
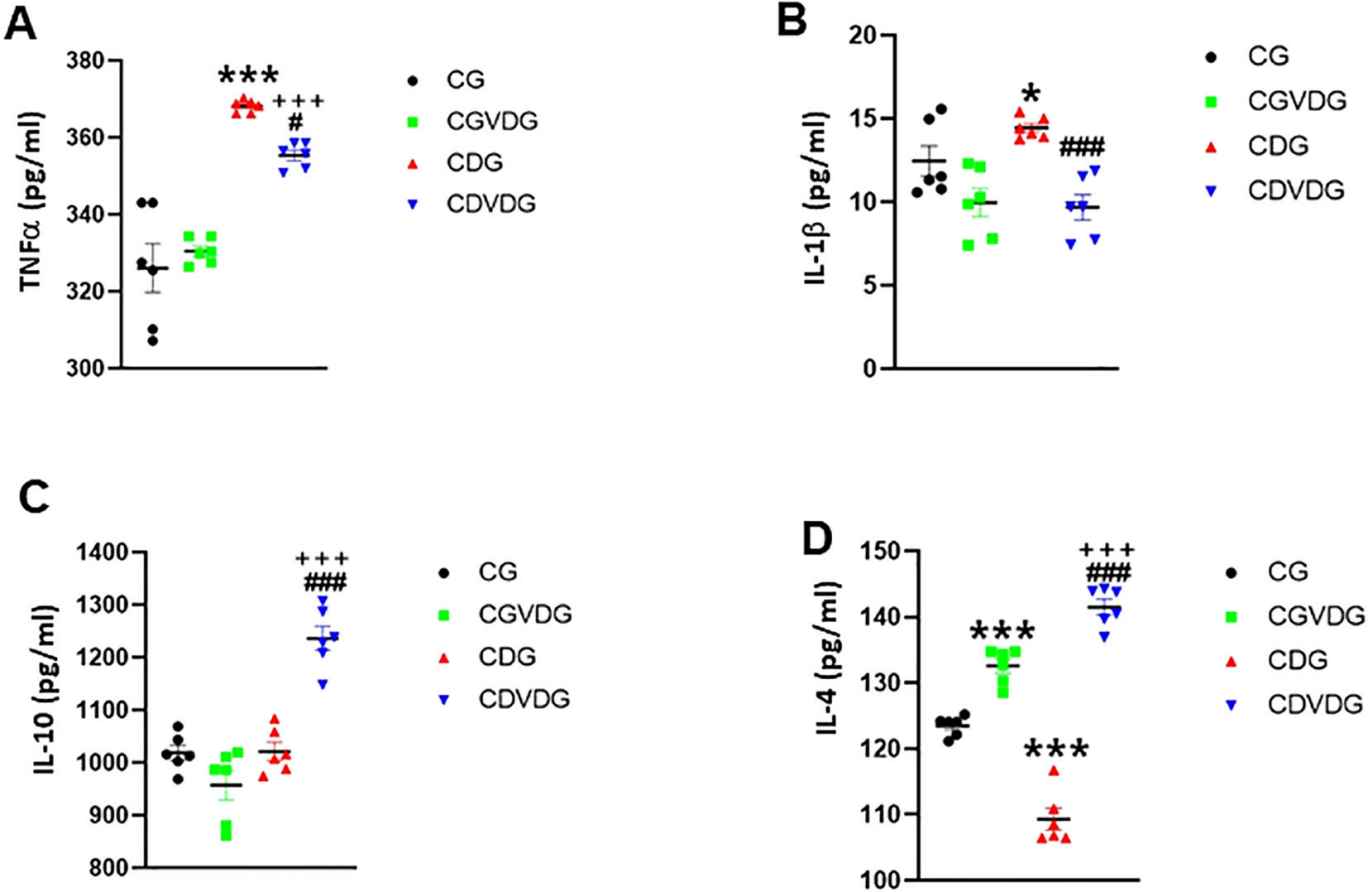
Effects of 1,25 VD3 supplementation on hepatic cytokine production in NASH rats. The proinflammatory, **(A)** TNF-α and **(B)** IL-1β levels, and the anti-inflammatory **(C)** IL-10 and **(D)** IL-4 levels in liver tissue lysates of indicated groups were measured using specific ELISA kits. Data are presented as mean ± SEM, n=6. *p,0.05 and ***p < 0.001 vs CG; #p < 0.05 and ###p < 0.001 vs CDG; +++p < 0.001 vs CG.

Given cytokine imbalance’s role in NASH, we measured the levels of liver TNF-α, IL-1β, IL-4, and IL-10 to elucidate pro-inflammatory and anti-inflammatory mechanisms. Pro-inflammatory cytokines TNF-α (**Figure 5A**) and IL-1β (**Figure 5B**) were elevated in the CDG (p < 0.001), but 1,25 VD3 supplementation significantly decreased TNF-α in the CDVDG (p < 0.05) and IL-1β in both CDVDG and CGVDG (p < 0.001). Conversely, anti-inflammatory cytokines IL-10 (**Figure 5C**) and IL-4 (**Figure 5D**) were increased in the CDVDG compared to the CGVDG (p < 0.001) and CDG (p < 0.001). Notably, IL-4 was elevated in the CGVDG compared to the CG (p < 0.001), but reduced in the CDG (p < 0.001). These findings suggest that 1,25 VD3 supplementation exerts anti-inflammatory effects in liver tissue, mitigating the pro-inflammatory consequences of the CDAA diet.

### 1,25 VD3 enhances anti-inflammatory CYP2J3/EET pathway and reduces pro-inflammatory sEH/DHET activity in NASH

**Figure 6:**
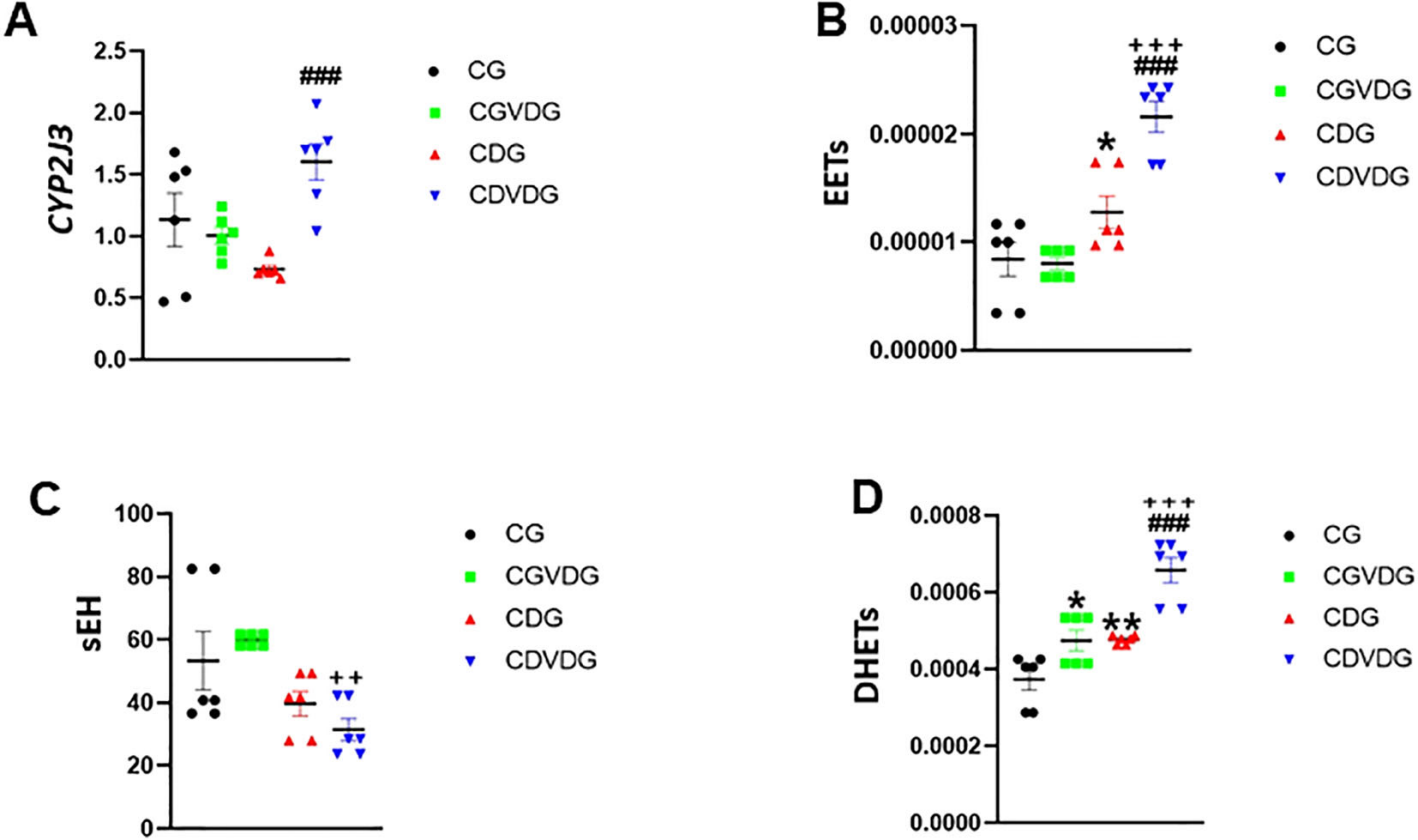
Effects of 1,25 VD3 supplementation on CYP450 and COX-2 pathways in liver of NASH rats. **(A)** CYP2J3 mRNA level was quantified by qPCR and expressed as relative mRNA levels following normalization to GAPDH mRNA levels. LC-MS/MS was used to measure **(B)** EETs, **(C)** sEH, and **(D)** DHETs levelss in livers of indicated groups. Data are presented as mean ± SEM, n=6. *p < 0.05 and **p < 0.01 vs CG; ###p < 0.001 vs CDG; ++p < 0.01.

Liver-expressed CYP2J3, a key epoxygenase, and the CYP450 enzyme modulates inflammation by converting AA into anti-inflammatory EETs and pro-inflammatory HETEs (8, 17). CYP2J3 levels were significantly higher in the CDVDG compared to the CDG (p < 0.01) (**Figure 6A**). CYP2J3 converts AA to anti-inflammatory EETs, particularly 5,6-EET, which was detected in liver tissue. Interestingly, EET levels were elevated in the CDG compared to the CG (p < 0.05), and remarkably enhanced in the CDVDG compared to both CG and CDG groups (all p < 0.001) (**Figure 6B**).

However, sEH, a key enzyme in the CYP 450 pathway, metabolizes EETs to pro-inflammatory DHETs. Our results showed that sEH activity was significantly lower in the CDVDG compared to the CGVDG (p < 0.01), suggesting reduced EET metabolism (**Figure 6C**). Conversely, DHETs (5,6-DHET, 8,9-DHET, 11,12-DHET, and 14,15-DHET) were detected, with significantly higher levels in the CDVDG compared to both CGVDG and CDG (both p < 0.001). Additionally, DHET levels were higher in the CGVDG compared to the CG (p < 0.05) (**Figure 6D**).

### Interactive effects of choline deficiency and 1,25 VD3 supplementation on liver function, oxidative stress, and inflammatory pathways in NASH

To explore the mechanisms behind NASH progression and the effects of 1,25 VD3 supplementation, we examined the interactive effects of the choline-deficient diet and 1,25 VD3 on liver and metabolic parameters (**Table 3**). A significant interaction indicates that the combined effects of the CDAA diet and 1,25 VD3 supplementation on liver and metabolic parameters were not simply the sum of their individual effects. Instead, the presence of one factor modified the influence of the other on the measured outcomes.

**Table 3:** Two-way ANOVA showing individual effects of the CD diet and 1,25 VD3 supplementation, as well as their interactions, across various biochemical and histopathological parameters.

Parameter	CD Diet Effect	1,25 VD3 Supplementation Effect	Interaction Effect (CD x 1,25 VD3)
Serum AST	Significant (p < 0.001)	Not significant (p > 0.05)	Significant (p < 0.001)
Serum ALT	Significant (p < 0.001)	Significant (p < 0.001)	Significant (p < 0.001)
Steatosis	Significant (p < 0.001)	Significant (p < 0.001)	Significant (p < 0.001)
Lobular inflammation	Significant (p < 0.001)	Significant (p < 0.001)	Significant (p < 0.001)
Ballooning degeneration	Significant (p < 0.001)	Significant (p < 0.001)	Significant (p < 0.001)
Activity Score (Histopathology)	Significant (p < 0.001)	Significant (p < 0.001)	Significant (p < 0.001)
SAF Score	Significant (p < 0.001)	Significant (p < 0.001)	Significant (p < 0.001)
MDA (Malondialdehyde)	Significant (p < 0.001)	Significant (p < 0.001)	Significant (p < 0.05)
TAOC (Total Antioxidant Capacity)	Significant (p < 0.001)	Significant (p < 0.001)	Significant (p < 0.001)
PPARα expression	Not significant	Not significant	No significant interaction
CTP-1 expression	Not significant	Not significant	No significant interaction
NF-κB levels	Not reported	Not reported	Significant (p < 0.05)
TNF-α levels	Significant (p < 0.001)	Not significant	Significant (p < 0.05)
IL-1β levels	Not significant	Significant (p < 0.001)	Not significant
IL-4 levels	Significant (p < 0.001)	Significant (p < 0.001)	Significant (p < 0.001)
IL-10 levels	Significant (p < 0.001)	Significant (p < 0.001)	Significant (p < 0.001)
M1/M2 Ratio	Significant (p < 0.001)	Significant (p < 0.001)	Significant (p < 0.001)
PPARγ expression	Not significant	Significant (p < 0.05)	Significant (p < 0.05)
CYP2J3 levels	Not significant	Tends to increase (p = 0.052)	No significant interaction
EETs levels	Significant (p < 0.01)	Significant (p < 0.01)	Significant (p < 0.01)
DHETs levels	Significant (p < 0.01)	Significant (p < 0.01)	Significant (p < 0.01)
sEH activity	Not significant	Not significant	Not significant

The interaction between the CDAA diet and 1,25 VD3 supplementation significantly affected various liver and metabolic parameters. 1,25 VD3 alone (CGVDG) did not affect liver enzyme levels, but its combination with the CDAA diet significantly altered both AST and ALT levels. Histopathological features such as steatosis, lobular inflammation, ballooning degeneration, activity score, and the SAF score were strongly influenced by both the CDAA diet and 1,25 VD3 supplementation, demonstrating a significant interaction between the two factors. The interaction between the CDAA diet and 1,25 VD3 supplementation significantly affected various liver and metabolic parameters. 1,25 VD3 alone (CGVDG) did not affect liver enzyme levels, but its combination with the CDAA diet significantly altered both AST and ALT levels. Histopathological features such as steatosis, lobular inflammation, ballooning degeneration, activity score, and the SAF score were strongly influenced by both the CDAA diet and 1,25 VD3 supplementation, demonstrating a significant interaction between the two factors.

Furthermore, markers of oxidative stress and antioxidant capacity, specifically MDA and TAOC, were significantly altered by the combined effects of both treatments. In terms of inflammatory markers, the interaction between the CDAA diet and 1,25 VD3 led to significant changes in NF-κB and TNF-α levels. Additionally, anti-inflammatory cytokines IL-4 and IL-10 were significantly influenced by this interaction, demonstrating significant enhancement in CDVDG group compared with CG. The M1/M2 macrophage ratio was significantly affected by both the CDAA diet and 1,25 VD3 supplementation, with the CDAA diet increasing the ratio (shifting toward M1) and 1,25 VD3 supplementation in the presence of the CDAA diet decreasing it (shifting toward M2), while 1,25 VD3 alone (CGVDG) had no effect, demonstrating a significant interaction between the CDAA diet and 1,25 VD3. Although the CDAA diet did not alter PPARγ expression, supplementation with 1,25 VD3 did, and the interaction between the two factors further influenced its expression. Moreover, both treatments affected the levels of EETs and DHETs, though no interaction was noted for sEH activity.

These findings underscore the complex interplay between choline deficiency and 1,25 VD3 supplementation, highlighting their combined effects on various liver functions, oxidative stress, inflammatory responses, and metabolic parameters.

### Correlation analysis of liver markers and metabolic parameters in response to 1,25 VD3 supplementation and the CDAA diet in NASH

Correlation analysis revealed significant associations between liver tissue variables and their correlated markers in response to 1,25 VD3 supplementation and the CDAA diet (**Table 4**). 1,25 VD3 supplementation mitigated the CDAA diet’s negative effects on liver, enhancing CYP2J3 expression and promoting positive correlations with EETs and DHETs. This supplementation also increased PPARγ expression, linked to anti-inflammatory effects, and reduced pro-inflammatory cytokines TNF-α and IL-1β. Elevated CYP2J3 levels correlated with lower EETs and MDA, suggesting potential benefits against oxidative stress. Conversely, PPARα expression positively correlated with TAOC and CTP-1, indicating enhanced antioxidant capacity and lipid transport. Both EETs and DHETs demonstrated positive correlations with IL-10, supporting their anti-inflammatory roles. In contrast, the CDAA diet elevated NF-κB and TNF-α levels, triggering in inflammatory responses. Importantly, DHETs’ positive correlation with PPARα, combined with 1,25 VD3’s benefits, underscores their critical role in counteracting the negative impacts of CDAA diet and regulating liver function and metabolic processes in NASH progression.

**Table 4:** Correlations between measured liver and metabolic variables and the effects of 1,25 VD3 and CDAA diet.

Measured Variable	Correlated Variable	Direction of Correlation	Effect of 1,25 VD3	Effect of CD Diet	r, p values
CYP2J3 expression	EETs	Negative	Increased	No effect	r=-0.790, p<0.01
CYP2J3 expression	MDA	Negative	Increased	No effect	r=-0.865, p<0.001
PPARα expression	TAOC	Positive	No effect	Increased	r=0.902, p<0.001; r=0.898, p<0.001
PPARα expression	CTP-1	Positive	No effect	Increased	r=0.908, p<0.001; r=0.854, p<0.001
NF-κB expression	MDA	Negative	Decreased	Increased	r=-0.748, p<0.01; r=-0.900, p<0.001
NF-κB expression	M1/M2 ratio	Negative	Decreased	Increased	r=-0.672, p<0.001; r=-0.826, p<0.01
TNF-α	MDA	Negative	Decreased	Increased	r=-0.727, p<0.01; r=-0.735, p<0.01
IL-1β	MDA	Negative	Decreased	No effect	r=-0.782, p<0.01; r=-0.629, p<0.05
PPARγ expression	IL-4	Positive	Increased	No effect	r=0.770, p<0.01; r=0.725, p<0.01
PPARγ expression	IL-10	Positive	Increased	No effect	r=0.585, p<0.05; r=0.585, p<0.01
EETs	IL-10	Positive	Increased	Increased	r=0.657, p<0.05
DHETs	MDA	Negative	Increased	Increased	r=-0.821, p<0.01
DHETs	NF-κB	Negative	Increased	Increased	r=-0.766, p<0.01
DHETs	TNF-α	Negative	Increased	Increased	r=-0.792, p<0.01
DHETs	M1/M2 ratio	Negative	Increased	Increased	r=-0.916, p<0.001
DHETs	TAOC	Positive	Increased	Increased	r=0.626, p<0.05
DHETs	PPARα	Positive	Increased	Increased	r=0.664, p<0.05
DHETs	CTP-1	Positive	Increased	Increased	r=0.627, p<0.05
sEH	TAOC	Negative	Increased	Increased	r=-0.707, p<0.01

• Measured variable: The primary variable measured in the study, related to liver function or metabolic processes.

• Correlated variable: Other variables found to correlate with the measured variable, based on statistical analysis.

• Direction of correlation: Indicates whether the correlation is positive or negative (e.g., an increase in one variable results in an increase or decrease in the other).

• Effect of 1,25 VD3: Whether 1,25 vitamin D3 supplementation had an effect on the measured variable.

• Effect of CDAA diet: Whether diet had an effect on the measured variable.

• r, p values: Statistical correlation (r) and significance (p) values indicating the strength and significance of the correlations between the measured and correlated variables.

## Discussion

In this study, we used a rat model of NASH induced by a CD diet to investigate the impact of 1,25 VD3. This active hormone improved liver function, enabling the liver to synthesize more 25 VD3, the precursor for 1,25 VD3. This suggests that alleviating liver dysfunction associated with the choline-deficient diet restores the liver’s capacity to produce 25 VD3. 1,25 VD3 supplementation also significantly modulated CYP450 metabolism, increasing CYP2J3 expression and EETs production. Additionally, 1,25 VD3 enhanced PPARα and CTP-1 expression, reduced MDA levels, and increased TAOC, thereby improving lipid peroxidation and antioxidant defenses in NASH rat livers. Furthermore, 1,25 VD3 supplementation exhibited potent anti-inflammatory effects by downregulating NF-κB and upregulating PPARγ. This led to a phenotypic shift in macrophages from pro-inflammatory M1 to anti-inflammatory M2, increasing IL-4 and IL-10 secretion while decreasing TNF-α production.

Expanding on the modulation of liver function and metabolic pathways by 1,25 VD3 (13), we further investigated its effects on AA metabolism, which has an important role in liver lipid peroxidation and inflammation in NASH. We investigated AA metabolism in NASH, given its established link to liver lipid peroxidation and inflammation. Previous studies have shown altered AA metabolites in NASH patients, reduced CYP2J2 expression in high-fat diet (HFD)-induced NASH mice (26), decreased EETs (15), and increased sEH (32) in NASH mice. Additionally, EETs and DHETs are elevated in NAFLD patients and MCD diet-induced NAFLD animal models (14, 27). In our study, we found increased EETs and DHETs, decreased sEH activity, and unchanged CYP2J3 expression in CD diet-induced NASH rats. These findings suggest that 1,25 VD3 supplementation modulates AA metabolism in NASH, favoring anti-inflammatory pathways through increased EETs and decreased sEH activity, which may contribute to its protective effects against liver damage and inflammation in NASH.

The mechanisms by which 1,25 VD3 improves lipid metabolism and mitigates inflammation are through PPARα and PPARγ signaling (3–5, 33–38). We observed that 1,25 VD3 upregulated PPARα and CPT-1, and shifted KC polarization. The shift in the composition of KCs from a pro-inflammatory phenotype (higher M1/M2 ratio) in the CDG group to a less inflammatory phenotype (lower M1/M2 ratio) in the CDVDG group likely contributed to the improved hepatic cytokine profile observed in the latter. This was reflected by reduced levels of pro-inflammatory cytokines (TNF-α and IL-1β) and increased levels of anti-inflammatory cytokines (IL-4 and IL-10) compared to the CDG group. Collectively, our results support the potential of 1,25 VD3 as an adjunct therapy for NASH, especially in individuals with vitamin D deficiency or insufficiency. However, further research is necessary to elucidate the optimal dosing regimens and long-term effects of 1,25 VD3 supplementation in NASH.

EETs, metabolites of the CYP450 pathway, have been shown to reduce liver lipid peroxidation and inflammation in NASH mice through PPARα and NF-κB signaling pathways (14, 15, 27, 39), highlighting their anti-inflammatory potential in various diseases (17–19). Our study extends these findings, demonstrating that 1,25 VD3 supplementation upregulates CYP2J3 expression, increases EETs and DHETs in rat liver tissue, and significantly interacts with NASH. This upregulation may contribute to anti-inflammatory effects, as CYP2J3 expression and EETs correlate with reduced lipid peroxidation markers (MDA, TAOC, PPARα, CTP-1) and inflammatory markers (NF-κB, M1/M2 ratio, TNF-α, IL-1β, IL-4, IL-10, PPARγ). Interestingly, DHETs exhibit anti-inflammatory activity, positively correlating with IL-10 and IL-4, which contrasts with previous reports of inactivity (40) or pro-inflammatory effects (20). However, recent studies reveal anti-inflammatory roles for DHETs in cystic pulmonary fibrosis (41) and pancreatic β-cell models (19), suggesting context-dependent effects. Furthermore, the context-dependent nature of DHETs’ effects highlights the need for further research to elucidate the specific mechanisms and conditions under which these molecules exert their effects. Elucidating these relationships will be crucial in harnessing the therapeutic potential of 1,25 VD3 supplementation and EETs/DHETs in NASH and other diseases characterized by lipid peroxidation and inflammation.

Study of interactive effects of 1,25 VD3 supplementation and the CD diet revealed significant impacts on liver function and metabolic markers. 1,25 VD3 mitigated CD diet-induced liver impairment by enhancing CYP2J3 expression, which correlated with higher EETs, DHETs, and reduced oxidative stress (lower MDA). Further correlation studies linked increased PPARγ expression with anti-inflammatory cytokines (IL-10, IL-4) and reduced pro-inflammatory markers (TNF-α, IL-1β), while PPARα expression correlated with improved antioxidant capacity (TAOC) and lipid transport (CTP-1). These findings emphasize the interactive effects of 1,25 VD3 supplementation and choline deficiency in favorably modulating liver function and inflammation.

Our study has some limitations. Recent research highlights the role of circadian rhythms in NAFLD (42–45), with vitamin D identified as a modulator of circadian regulation (46, 47). However, we did not study circadian-related markers such as CLOCK, BMAL1, CRY, and PER1/2. Instead, we focused on metabolic proteins, metabolites, and CYP450 enzymes to investigate how 1,25 VD3 improves lipid peroxidation and inflammation via the CYP450 and AA pathways, which are critical in NASH progression. Secondly, liver weight and treatment-induced changes in cellular composition were not considered when interpreting cytokine levels. However, the shifts in KC composition likely influenced cytokine modulation and macrophage polarization, as discussed. Future studies could investigate the contributions of individual liver cell types to cytokine modulation. Thirdly, while we assessed NF-κB expression using Western blotting, we acknowledge that immunohistochemistry (IHC) could have been used to validate protein localization and spatial distribution. However, since the antibody detects additional bands besides the 65 kDa NF-κB, Western blotting allows for more accurate measurement of NF-κB expression than IHC.

Collectively, our study revealed novel mechanisms by which 1,25 VD3 supplementation may serve as a valuable adjunct therapy for NASH management, particularly in patients with vitamin D deficiency or insufficiency. Further research is necessary to elucidate the optimal dosing regimens and long-term effects of 1,25 VD3 supplementation in NASH.

## Data Availability

The original contributions presented in the study are included in the article/supplementary material. Further inquiries can be directed to the corresponding authors.
